# Rare Case of Systemic Cryptococcal Lymphadenopathy in a Persian Cat

**DOI:** 10.1002/vms3.70564

**Published:** 2025-09-02

**Authors:** Chang‐Hyeon Choi, Keon Kim, Chang‐Yun Je, Kwang‐Jun Lee, Woong‐Bin Ro, Chang‐Min Lee

**Affiliations:** ^1^ Department of Veterinary Internal Medicine College of Veterinary Medicine Chonnam Nation University Gwangju South Korea; ^2^ College of Veterinary Medicine and BK21 FOUR Program Chonnam National University Gwangju Republic of Korea; ^3^ Division of Zoonotic and Vector Borne Disease Research, National Institute of Health Korea Disease Control and Prevention Agency Cheongju Republic of Korea

**Keywords:** cats, cryptococcosis, lymphadenopathy, retinal detachment

## Abstract

**Background:**

Cryptococcosis is the most common systemic fungal infection in cats, typically presenting with respiratory or neurological signs. However, cases without these hallmark symptoms are rare and often misdiagnosed. This case is noteworthy for its atypical presentation, where a cat developed systemic cryptococcosis without the typical respiratory or neurological involvement, making it particularly challenging to diagnose. In addition, the overlap of clinical signs with other diseases like lymphoma underscores the importance of considering cryptococcosis in differential diagnoses.

**Case presentation:**

A 7‐year‐old castrated male Persian cat was referred due to generalized cutaneous nodules, including a prominent 4–5 cm nodule on the neck, and generalized lymphadenopathy. Neurological examination revealed an absent menace response in the left eye, raising suspicion of vision loss. Blood tests indicated hyperglobulinemia and the presence of medium‐to‐large lymphoid cells in the peripheral blood. Diagnostic imaging showed systemic lymphadenopathy, retinal detachment and minimal ascites. Fine needle aspiration of the lymph nodes revealed yeast‐like organisms, and culture confirmed *Cryptococcus neoformans*. Initially treated with itraconazole, the therapy was switched to fluconazole due to better CNS penetration. The lymph node enlargement improved within one week, but further follow‐up was limited due to owner constraints.

**Conclusions:**

This case emphasizes the diagnostic challenges posed by atypical presentations of feline cryptococcosis, particularly in the absence of respiratory and neurological symptoms. It highlights the importance of considering systemic fungal infections in the differential diagnosis of lymphadenopathy. The overlap with diseases like lymphoma further underscores the need for comprehensive diagnostic workups, including cytology and culture, to ensure accurate diagnosis and effective treatment.

## Graphical abstract

A rare case of systemic feline cryptococcosis with lymphadenopathy and retinal detachment, but without respiratory or neurological signs. Diagnosis was confirmed by cytology and culture.

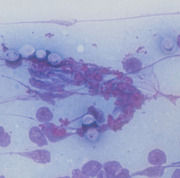

**Abbreviations**
CLAT: Cryptococcus latex agglutination test
CNS: Central nervous system
FeLV: Feline leukemia virus
FIP: Feline infectious peritonitis
FIV: Feline immunodeficiency virus
FNA: Fine needle aspiration
OS: Left eye
PCR: Polymerase chain reaction
PLR: Pupillary light reflex

## 1 Background

*Cryptococcus neoformans* is a globally distributed, yeast‐like fungus that commonly affects immunocompromised individuals and animals, particularly cats (Kwon‐Chung et al. 2014). This fungus is characterized by a thick polysaccharide capsule that aids in immune evasion and reproduces via narrow‐based budding (Doering 2009). Transmission primarily occurs through inhalation of desiccated yeast cells or spores from contaminated environments, such as bird droppings or decaying vegetation, with the nasal cavity being the most common site of initial infection (Malik et al. 1997).

Cryptococcosis is the most prevalent systemic fungal disease in cats (Trivedi, Sykes, et al. 2011). Although the nasal form of cryptococcosis is most commonly reported and typically presents with chronic nasal discharge and sneezing, ocular manifestations—including anterior uveitis and chorioretinitis—can also occur, often linked to central nervous system (CNS) involvement (Trivedi, Malik, et al. 2011b). In rare cases, the disease may affect the lower urinary tract or result in systemic lymphadenopathy. Systemic cryptococcosis occurs through haematogenous dissemination. This can lead to signs such as meningoencephalomyelitis, uveitis, chorioretinitis, osteomyelitis, polyarthritis, systemic lymphadenitis or multi‐organ involvement, including the kidneys. Notably, the systemic form may develop with or without the classic presentation of nasal disease (Pennisi et al. 2013).

Feline cryptococcosis is less frequently reported in Asia compared to regions like Australia, Canada and the United States, where it is more prevalent due to environmental exposure to Cryptococcus species (C. R. O'Brien et al. 2006). In Asia, the majority of cryptococcosis cases involve *C. neoformans* var. grubii and are typically linked to immunocompromised individuals, and rarely affect healthy cats (Khayhan et al. 2013). While *Cryptococcus* species are present in Asia, reports of feline cryptococcosis are relatively rare, likely due to regional environmental and epidemiological differences (Khayhan et al. 2013).

This report introduces a unique clinical presentation of systemic cryptococcosis in a cat without the typical clinical signs. Nasal involvement is the most common presentation, and disseminated or atypical symptoms without respiratory or neurological signs are rare (C. O'Brien et al. 2004). Furthermore, this case shares clinical similarities with feline lymphoma, emphasizing the need to include cryptococcosis in the differential diagnosis of cats presenting with lymphadenopathy.

## 2 Case Presentation

A 7‐year‐old castrated male Persian cat was referred to our veterinary teaching hospital at Chonnam National University with generalized cutaneous nodules, including a large 4–5 cm nodule on the neck. Due to generalized lymphadenopathy, lymphoma was considered as a differential diagnosis by the referring local animal hospital. Upon physical examination at our hospital, no cutaneous lesions were observed. However, bilateral enlargement of the submandibular, popliteal and inguinal lymph nodes was noted. During the neuro‐ophthalmological examination, the menace response in the left eye (OS) was absent, raising suspicion of vision loss; however, the pupillary light reflex (PLR) was normal, and no other notable findings were observed. The systolic blood pressure was measured at 135 mmHg, which was within the normal range.

**FIGURE 1:**
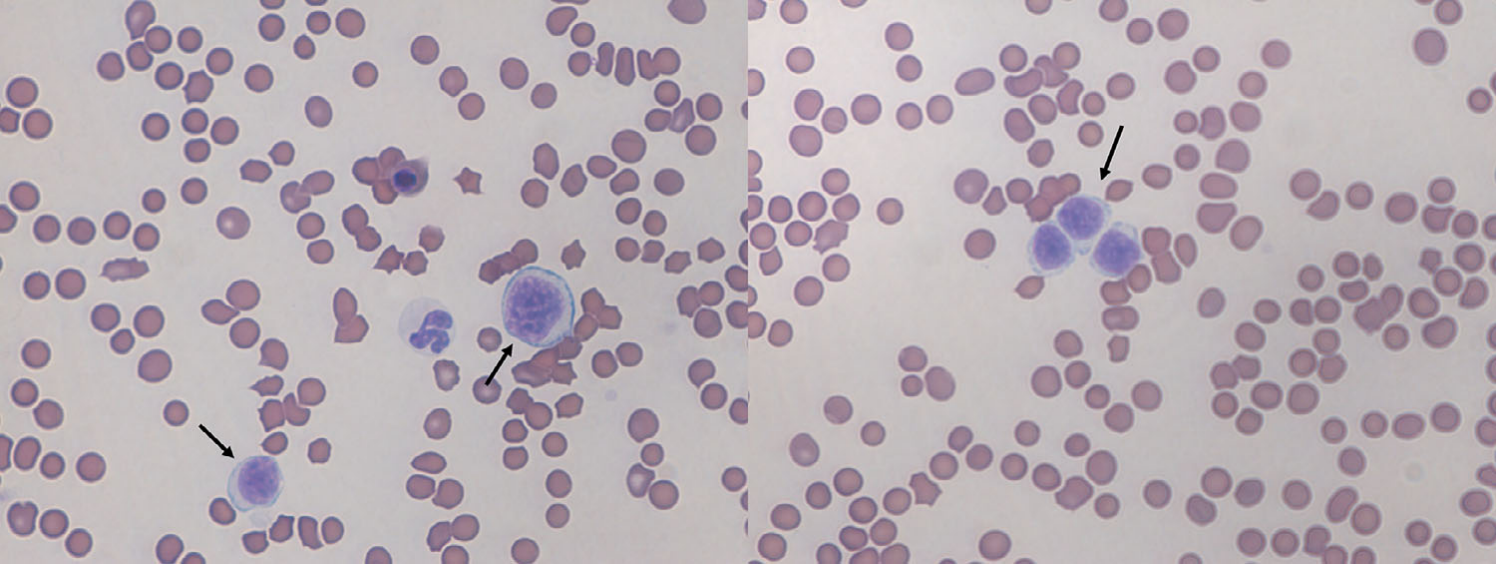
Peripheral blood smear. Multiple medium‐to‐large lymphoid cells (black arrow) were identified (Diff‐Quik stain, 1000× magnification).

The complete blood count (ProCyte Dx, IDEXX Laboratories, Inc., USA) and serum chemistry (Catalyst One, IDEXX Laboratories, Inc., USA) revealed decreased haematocrit (25%; reference range, 30.3%–52.3%) and hyperglobulinaemia (5.6 g/dL; reference range, 2.8–5.1 g/dL), with a low albumin‐to‐globulin ratio (0.6). In addition, multiple medium‐to‐large lymphoid cells were identified on the peripheral blood smear (Figure 1).

Serological tests (SNAP FIV/FeLV Combo, IDEXX Laboratories Inc., USA) for feline leukaemia virus (FeLV) and feline immunodeficiency virus (FIV) were negative. Polymerase chain reaction (PCR) testing for *Calicivirus*, *Influenza virus type A*, *Reovirus*, *Feline herpesvirus*, *Chlamydophila felis*, *Mycoplasma felis* and *Bordetella bronchiseptica* also yielded negative results. To rule out feline infectious peritonitis (FIP), real‐time PCR, an antibody test kit (ImmunoComb Feline Coronavirus (FCoV) [FIP] Antibody Test Kit, Biogal–Galed Laboratories, Acs Ltd., Israel) and the Rivalta test were performed, all of which were negative.

**FIGURE 2:**
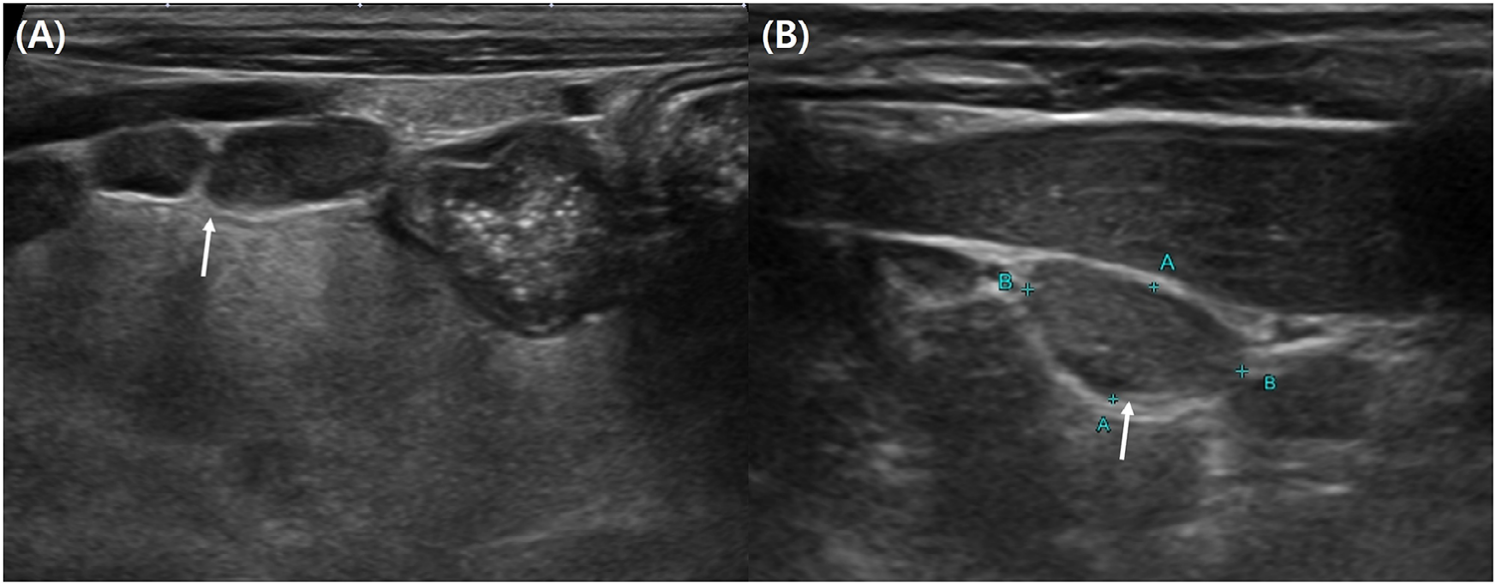
Abdominal ultrasound with a 12 MHz linear transducer (PLT‐1204BT, Canon Medical Systems Corp., Japan). Jejunal (A) and splenic (B) lymph node (white arrow) enlargement was shown.

**FIGURE 3:**
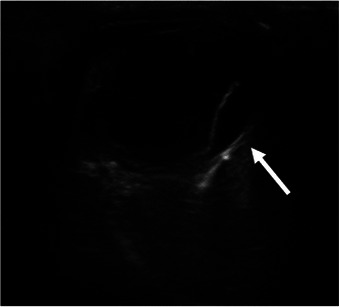
Ocular ultrasound with a 12 MHz linear transducer (PLT‐1204BT, Canon Medical Systems Corp., Japan). Left eye (OS) retinal detachment (white arrow) was suspected.

X‐rays revealed an enlarged sternal lymph node. Abdominal ultrasound (Aplio 500, Canon Medical Systems Corp., Japan) showed generalized celiac lymph node enlargement, including the jejunal and splenic lymph nodes (Figure 2). Only a small amount of ascites was detected, allowing for the collection of a very small sample. Ocular ultrasound (Aplio 500, Canon Medical Systems Corp., Japan) of the OS revealed retinal detachment (Figure 3).

**FIGURE 4:**
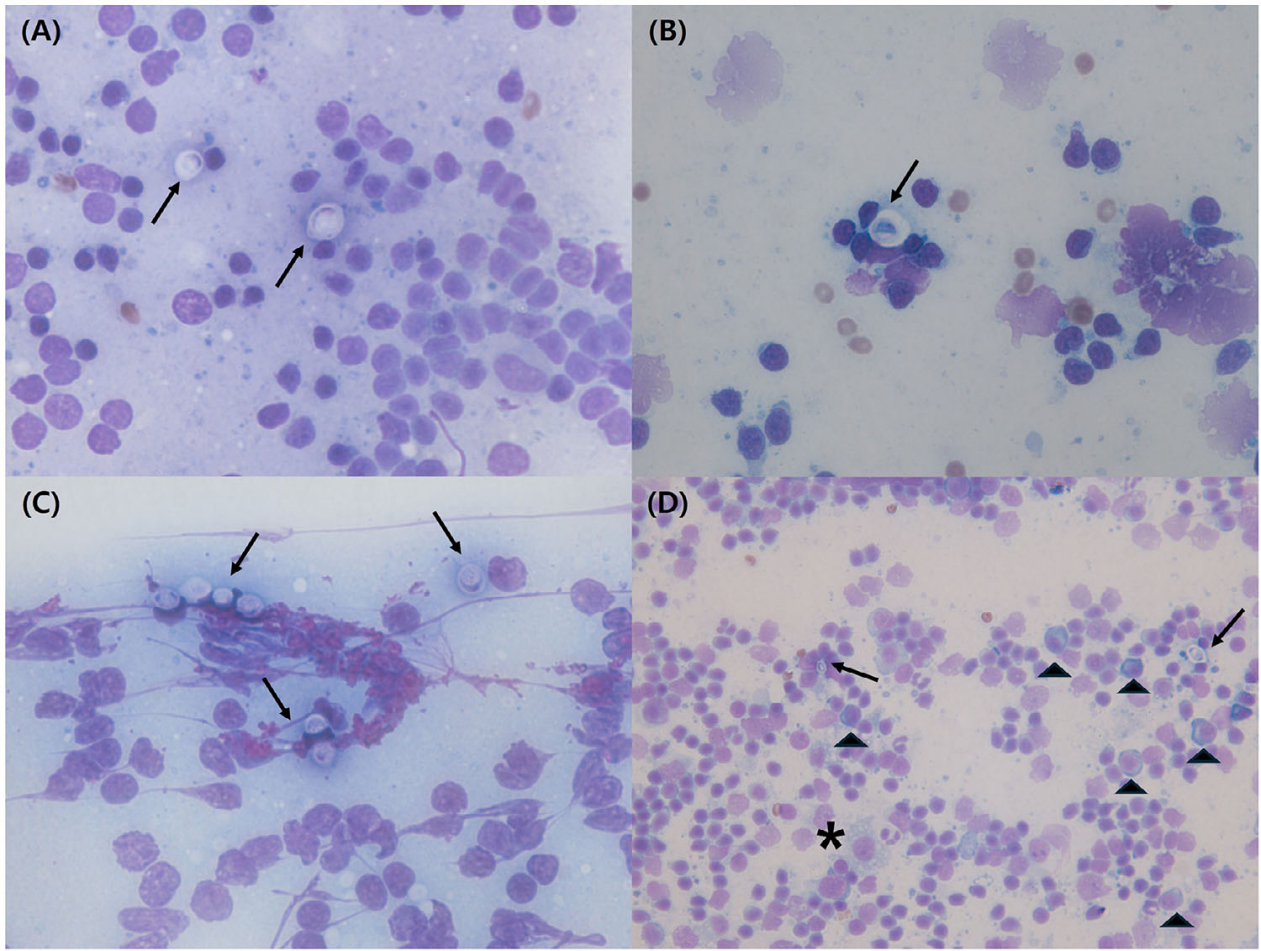
Fine needle aspiration of lymph node (Diff‐Quik stain). Multiple globular, encapsulated cells (black arrow), plasma cell (black arrow head), and macrophages (asterisk) were identified in inguinal (1000× magnification) (A), popliteal (1000× magnification) (B), submandibular (1000× magnification) (C), and splenic (400× magnification) (D) lymph node.

Fine needle aspiration (FNA) of the palpable lymph nodes (inguinal, popliteal and submandibular), as well as the splenic lymph nodes, was performed for cytological evaluation. In all sites, small lymphocytes were predominant, and globular, encapsulated materials, suspected of being yeast, were identified. In addition, plasma cells and macrophages were observed in the splenic lymph node (Figure 4).

As a fungal infection was suspected, a culture test was requested using FNA samples from the lymph nodes, and itraconazole (Sinsin Itraconazole Tab., SinSin Pharm Co., Ltd., South Korea) (5 mg/kg, q24hr, PO) was preemptively administered. The culture test confirmed the presence of *C. neoformans*. Given the systemic lymph node enlargement, retinal detachment, and the culture test result, the patient was diagnosed with systemic cryptococcosis. Following definitive diagnosis, itraconazole was substituted with fluconazole (Diflex Cap., Dong‐A ST Co., Ltd., South Korea) (5 mg/kg, q12hr, PO). The lymph node enlargement initially improved within one week of itraconazole treatment. However, further follow‐up could not be conducted due to financial constraints and the clinic's distant location.

## 3 Discussion and Conclusion

This case highlights atypical clinical presentations of feline cryptococcosis, which can be challenging to diagnose. The absence of respiratory signs, combined with systemic lymphadenopathy, retinal detachment and large lymphoid cells in the peripheral blood, represents a rare clinical presentation. This emphasizes the need for comprehensive diagnostic workups in such cases, including advanced cytological analysis and the use of additional tests like flow cytometry and immunostaining to differentiate between infectious and neoplastic conditions (Reggeti and Bienzle 2011). Since the diagnosis was confirmed via culture, additional tests were not required in this case (Pennisi et al. 2013). Moreover, it underscores the importance of considering systemic cryptococcal infections in the differential diagnosis of patients with lymphadenopathy, even when neoplastic diseases are suspected and common respiratory symptoms are absent.

Cryptococcosis in cats is typically diagnosed via cytology, as the yeast‐like fungus *C. neoformans* often appears in large numbers in affected tissues, making it relatively easy to identify by its characteristic structure (Pennisi et al. 2013). However, it is important to note that a negative cytology result does not completely exclude the diagnosis of cryptococcosis, particularly in cases where the fungal presence is minimal or the sampled lesion is not representative (Pennisi et al. 2013). In such instances, diagnosis can be challenging. Additional tests, such as the cryptococcus latex agglutination test (CLAT), serotyping, PCR and culture, can aid in the diagnostic process (Pennisi et al. 2013). In this case, cytological evidence of lymphadenitis was confirmed in the systemic lymph nodes, and although yeast infection was suspected, the absence of respiratory or neurological signs of cryptococcosis made the initial diagnosis challenging. Therefore, a definitive diagnosis was achieved through culture testing, enabling the implementation of appropriate treatment. 

In most feline cases, cryptococcosis typically presents with nasal cavity involvement, often manifesting as sneezing and nasal discharge (Trivedi, Malik, et al. 2011b). However, in this case, no respiratory symptoms were observed, which is unusual since the nasal cavity is typically affected before systemic dissemination. Although rare cases without respiratory signs have been reported, they usually involve neurological symptoms, also absent in this case (Trivedi, Malik, et al. 2011a). The retinal detachment observed in this patient was likely caused by cryptococcal infection, as it was consistent with previously reported ocular involvement in disseminated cryptococcosis (Trivedi, Malik, et al. 2011b; Trivedi, Malik, et al. 2011a). Furthermore, systemic hypertension, a common cause of retinal detachment in cats, was excluded, as the systolic blood pressure was within the normal range (Gelatt and Mackay 2000). However, the aetiology could not be definitively confirmed due to the lack of further ophthalmic testing.

The patient presented with generalized lymphadenopathy, which initially led to the consideration of lymphoma as a differential diagnosis (Malik et al. 2003). However, distinguishing reactive lymphocytes from neoplastic lymphocytes based solely on morphological features can be challenging. Reactive lymphocytes, often observed in cases of systemic immune stimulation, may resemble lymphoblasts, complicating the staging or diagnosis of conditions like lymphoma. While lymphoblasts are rarely seen in the peripheral blood of healthy cats, they may appear in response to strong immune stimulation (Valenciano et al. 2013). In this case, the presence of medium‐to‐large lymphocytes in the peripheral blood, suspected to be reactive, complicated the diagnosis as they can resemble neoplastic lymphocytes, making it difficult to exclude conditions like lymphoma.

In the treatment of feline cryptococcosis, both itraconazole and fluconazole are commonly used antifungal agents, but fluconazole may offer certain advantages. Studies have shown that fluconazole is associated with shorter treatment durations and fewer relapses compared to itraconazole, particularly in cases where long‐term maintenance therapy is required (Saag et al. 1999; Stott et al. 2018). Moreover, fluconazole has better penetration into the CNS, making it more effective in managing cases involving neurological involvement. While itraconazole remains effective, especially in severe or refractory cases, fluconazole's more favourable pharmacokinetics and shorter treatment time make it a preferred choice in many cases (Saag et al. 1999; Stott et al. 2018). Based on these findings, we decided to switch from itraconazole to fluconazole in this case to optimize treatment outcomes as the client had difficulty administering the medication.

Nonetheless, several limitations need to be addressed. First, due to the owner's decision to discontinue follow‐up visits because of financial concerns and the long distance to the clinic, the prognosis could not be evaluated. In addition, other differential diagnoses, such as FIP, lymphoma and leukaemia, were not completely ruled out. Although real‐time PCR for FIP was performed and returned a negative result, it was conducted using EDTA whole blood due to the lack of ascites, which has a lower sensitivity (Doenges et al. 2017). Finally, as cryptococcosis is a zoonotic disease, the cultured samples were not released by the testing facility due to quarantine regulations, preventing the additional PCR test for serotyping from being conducted.

In conclusion, this case highlights the diagnostic challenges of systemic cryptococcosis with atypical presentations and reinforces the importance of considering it in the differential diagnosis of lymphadenopathy, particularly when typical signs such as respiratory or neurological symptoms are absent. While culture testing proved sufficient for diagnosis in this case, incorporating advanced diagnostic techniques remains crucial for differentiating infectious from neoplastic conditions.

## Author Contributions

**Chang‐Hyeon Choi**: data curation, formal analysis, investigation, visualization, writing – original draft. **Keon Kim**: data curation, formal analysis, investigation, visualization, writing – original draft. **Chang‐Yun Je**: data curation, formal analysis, investigation, visualization, writing – original draft. **Kwang‐Jun Lee**: conceptualization, funding acquisition, project administration, resources, supervision, validation, writing — review and editing. **Woong‐Bin Ro**: conceptualization, funding acquisition, project administration, resources, supervision, validation, writing – review and editing. **Chang‐Min Lee**: conceptualization, funding acquisition, project administration, resources, supervision, validation, writing – review and editing.

## Ethics Statement

This study did not require ethical approval because it did not involve any interventions and utilized previously collected animal tissues obtained as part of diagnostic pathology procedures.

## Consent

Consent was obtained from the present owner of the dog for publication of this case report and any accompanying images.

## Conflicts of Interest

The authors declare no conflicts of interest.

## Peer Review

The peer review history for this article is available at https://www.webofscience.com/api/gateway/wos/peer‐review/10.1002/vms3.70564.

## Data Availability

The data that support the findings of this study are available from the corresponding author upon reasonable request.
